# Publication of the Transactions of the Medical Association of Georgia

**Published:** 1886-05-20

**Authors:** 


### PUBLICATION OF THE TRANSACTIONS OF THE MEDICAL ASSOCIATION OF GEORGIA.

It will be remembered by our readers that at the previous meeting of the Association, in Savannah (April, 1885), it was voted to publish the Transactions in the *Atlanta Medical and Surgical Journal*—the secretary, who was the editor of the Journal, being thus gratuitously furnished with original matter for his journal for a whole year, and the members of the Association placed in a position where they must subscribe for that journal or do without the Transactions.

When this action was taken there was but a small number of members present, and no one connected with the SOUTHERN MEDICAL RECORD was there to enter a protest, and no one seemed to have thought of the injustice to our journal and of the inexpediency of such a measure. As soon as we became aware of the action thus taken, we protested against it as unjust to our journal, to our subscribers who are members of the Association, and to the profession at large.

The RECORD has been a leading advocate, and organ, of the profession for long years, pledged to vindicate the honor and rights of its members at a time when there was no other well-established journal in the State. We were appealed to to protest against the measure as calculated to divide the profession and break down the Association. The injustice of the measure was so apparent that it attracted the attention of many of our subscribers; and one of our exchanges in Virginia—the *Southern Clinic*—referred to it under the caption of "Discrimination with a vengeance," stating that a similar measure had been tried in that State, and with bad results—destroying the harmony of the Association, and leading to much bitter controversy. The final result was, after five years of discord, that the journal plan was abandoned, and the Transactions ordered to be published in pamphlet form, and let out to the lowest bidder. Under this contract plan the Transactions are now published in that State at a cost of

*about one-third the amount which the Georgia Association has been paying!*

This was one of the points we had intended to show at the late meeting of the State Association had not the publishing committee voluntarily recommended a return to the old method of publication. We also were prepared to show by private letters in our possession a strong opposition existing in the profession to the measure. We had further designed to introduce a resolution to adopt the Virginia plan of letting out the contract for printing the Transactions to the lowest bidder, as a measure of economy, and with a view to reduce the annual dues, but being called away before the close of the Association, we failed to do so. It is important, however, for the publication committee, and we think it is clearly their duty to let out the publication of the Transactions to the lowest bidder, and we trust they will do so.

We are glad that the recommendation of the committee to return to the former method of publication was unanimously adopted, because we have never sought to take any unfair advantage, or to do anything calculated to injure the Medical Association or any of its members. We have never sought to sustain error nor to urge a wrong measure by ways and means that were more to be deprecated than the measure itself. We have uniformly, as the RECORD will show, acted upon principle and endeavored to uphold the honor and dignity of the profession, by opposing error in a bold and independent manner, and in strict accordance with the laws which govern the profession. In pursuing this course we have encountered opposition and incurred the displeasure of a few; yet we are glad to be able to say that in *every controversy* in which we have thus encountered opposition and enmity we have ultimately been sustained in *the principles* for which we contended. In saying this, our oft-repeated opinion that the profession of Georgia are honorable and true, and will do right when properly informed, is, in this case as in others, fully sustained.

It has been our custom to teach the strict and rigid observance of Principle, as a medical lecturer, and especially have we sought to guard the student from sustaining error merely to be consistent with former action or to gratify friends. We have known medical men to injure their reputations and prospects in the profession by adhering to friends in error, and allowing themselves to become prejudiced against men whom, in their inmost hearts, they knew to be true and incapable of knowingly sustaining or committing a wrong. We have known ministers of the Gospel to preach the Truth with great power in the pulpit who neutralized the results of their own teaching, and destroyed their influence for good, by refusing to condemn the wrong in matters of controversy wherein a friend was implicated, even though great and important principles of truth were thereby ignored and error and injustice encouraged.

Politicians often prove themselves destitute of true patriotism, and unworthy the support of honest men, by ignoring great constitutional principles and favoring measures solely for the purpose of supporting or shielding the wrongs of personal or partisan friends. The time has come when men of this stamp should no longer be sustained, whether in the ecclesiastical, political or medical departments. Let all good and true men stand by the right and the laws by which we profess to live, and not be influenced to abandon principle, and to cease their efforts for good because of the opposition and criticisms of those who seek to aggrandize themselves by pulling down other and better men.

We have written these thoughts at the request of friends, and as journalists having at heart the honor and good of the profession of Georgia, which, though ever ready to vindicate, we know as journalists, has been injured in the house of its friends, and which does not sustain the high reputation abroad to which it is justly entitled.

